# Recovery of new-onset kidney disease in COVID-19 patients discharged from hospital

**DOI:** 10.1186/s12879-021-06105-8

**Published:** 2021-04-29

**Authors:** Nan-Hui Zhang, Yi-Chun Cheng, Ran Luo, Chun-Xiu Zhang, Shu-Wang Ge, Gang Xu

**Affiliations:** Department of Nephrology, Division of Internal Medicine, Tongji Hospital, Tongji Medical College, Huazhong University of Science and Technology, No.1095 Jiefang Road, Wuhan, 430030 Hubei China

**Keywords:** COVID-19, Kidney disease, Renal recovery

## Abstract

**Background:**

Coronavirus disease 2019 (COVID-19) has emerged as a major global health threat with a great number of deaths worldwide. Despite abundant data on that many COVID-19 patients also displayed kidney disease, there is limited information available about the recovery of kidney disease after discharge.

**Methods:**

Retrospective and prospective cohort study to patients with new-onset kidney disease during the COVID-19 hospitalization, admitted between January 28 to February 26, 2020. The median follow-up was 4 months after discharge. The follow-up patients were divided into the recovery group and non-recovery group. Descriptive statistics and between-groups comparison were used.

**Results:**

In total, 143 discharged patients with new-onset kidney disease during the COVID-19 hospitalization were included. Patients had a median age was 64 (IQR, 51–70) years, and 59.4% of patients were men. During 4-months median follow-up, 91% (130 of 143) patients recovered from kidney disease, and 9% (13 of 143) patients haven’t recovered. The median age of patients in the non-recovery group was 72 years, which was significantly higher than the median age of 62 years in the recovery group. Discharge serum creatinine was significantly higher in the non-recovery group than in the recovery group.

**Conclusions:**

Most of the new-onset kidney diseases during hospitalization of COVID-19 patients recovered 4 months after discharge. We recommend that COVID-19 patients with new-onset kidney disease be followed after discharge to assess kidney recovery, especially elderly patients or patients with high discharge creatinine.

**Supplementary Information:**

The online version contains supplementary material available at 10.1186/s12879-021-06105-8.

## Background

The ongoing coronavirus disease 2019 (COVID-19) is a worldwide pandemic caused by severe acute respiratory syndrome coronavirus 2 (SARS-CoV-2) [1, 2]. Up to November 28, 2020 the World Health Organization (WHO) recorded 60,534,526 confirmed cases and 1,426,101 deaths in 216 countries worldwide [3].

COVID-19 is a respiratory infectious disease that primarily causes pneumonia and severe hypoxemia, but the lungs are not the only organ affected. Reports have confirmed that SARS-CoV-2 can invade cells via angiotensin-converting enzyme (ACE2) [4, 5] and that ACE2 is highly expressed in the human kidney. Moreover, relevant autopsy data from COVID-19 patients found clusters of coronavirus particles with distinct spikes present in renal tubular epithelial cells [6], suggesting that the kidney may be the target of SARS-COV-2. In addition, available data indicated the high incidence of kidney disease in COVID-19 patients. Previous studies showed that 43.9% of patients with COVID-19 had proteinuria on admission, while 14.4% of patients were admitted with elevated blood creatinine [7]. Furthermore, it is reported 65.8% of patients experienced remission of proteinuria during hospitalization [8]. However, the studies performed to date have been limited to observations during hospitalization, so the recovery of kidney disease remains unknown in COVID-19 patients who survive after hospitalization.

In the present study, we aimed to describe the incidence and short-term recovery of new-onset kidney disease during hospitalization in COVID-19 patients who discharged alive.

## Methods

### Study design and patients

This was a retrospective and prospective study conducted in Tongji hospital, located in Wuhan, one of the main tertiary teaching hospitals, which was assigned responsibility for the treatments of severe COVID-19 patients by the local government. We included all patients who tested positive by polymerase chain reaction testing of a nasopharyngeal sample for COVID-19, developed new kidney disease during hospitalization, admitted to the Tongji hospital, and were discharged alive from January 28 to February 26, 2020 (*N* = 865). We excluded the following patients: (1) those aged < 18 years, (2) those who died during hospitalization, (3) those who were dialysis patients, (4) those who were renal allograft recipients, and (5) those with previous chronic kidney disease (CKD), based on careful and repeated history taking. All discharged patients met the uniform discharge criteria of the Chinese clinical guidance for the diagnosis and treatment of COVID-19 pneumonia issued by the National Health Commission (the absence of fever for at least 3 days, substantial improvement in both lungs in chest computed tomography, clinical remission of respiratory symptoms, and two nasal and pharyngeal swab samples negative for SARS-CoV-2 RNA obtained at least 24 h apart) [9].

**Fig. 1:**
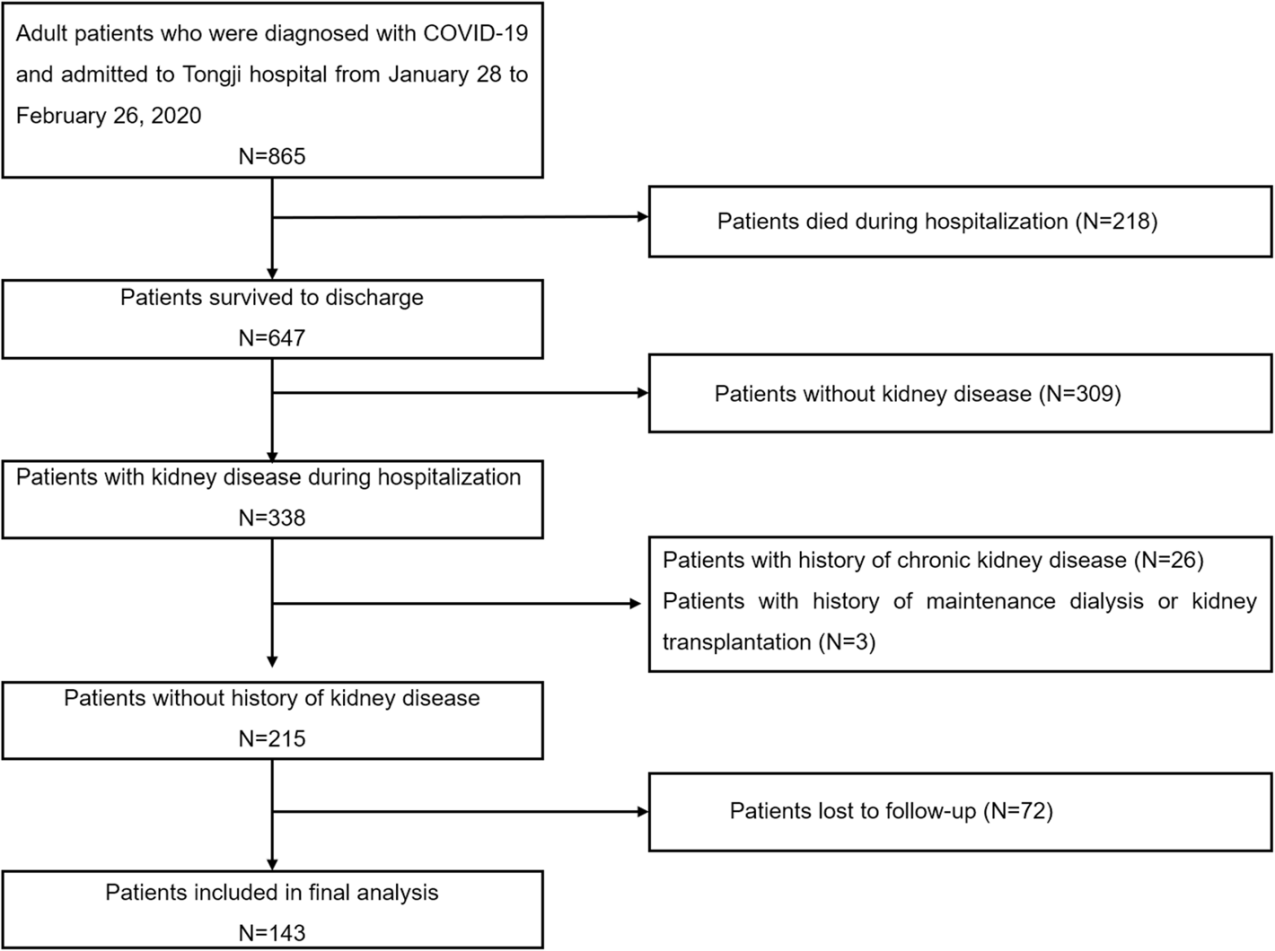
Flow chart of the selection of eligible subjects

All those patients included in the present study were re-contacted after discharge from the hospital to determine their willingness to repeat renal laboratory and urine dipstick tests. Of the 215 patients enrolled in the present study, 143 were available for follow-up (Fig. 1), and 72 patients were lost to follow-up because they declined to participate or did not recheck urine analysis. Therefore, 143 patients finally met the screening criteria and had follow-up data. We compared the characteristics of the 72 patients lost to follow-up and 143 patients with follow-up. It showed no significant difference existing between the two groups (Supplemental Table 1).

Individual-level informed consent was not obtained given the retrospective nature of the analysis of an electronic medical record. The study protocol and waived written informed consent was approved by the Medical Ethics Committee of Tongji Hospital (No.TJ-C20200132). All methods were carried out in accordance with relevant guidelines and regulations.

### Data collection and measurement

The demographic characteristics, clinical symptoms, laboratory data were extracted from electronic medical records. All the comorbidities were reported by patients or family members. Venous blood samples were collected from all participants who attended for follow-up. Serum creatinine, blood urea nitrogen, hemoglobin, leukocyte count, lymphocyte count, platelet count, examination of D-dimer and high-sensitivity C-reactive protein were measured. Urine dipstick test to assess for proteinuria was performed in all patients. The estimated glomerular filtration rate (eGFR) was calculated with Chronic Kidney Disease Epidemiology Collaboration (CKD-EPI) equation [10]. The baseline clinical characteristics and laboratory data were ascertained at the time of admission. The data were reviewed by a trained team of physicians. This has been described in detail in previous studies [11].

The method for detecting SARS-CoV-2 in COVID-19 patients has been described in detail elsewhere [8]. In order to extract SARS-Cov-2 RNA, throat swab samples were collected from patients with possible COVID-19 pneumonia. The respiratory sample RNA isolation kit (Biogerm, Shanghai, China)was used to extract nucleic acids. And SARS-CoV-2 detection kit (Biogerm, Shanghai, China) was used to detect the ORFlab gene (nCovORFlab) and the N gene (nCoV-NP) according to the manufacturer’s instructions, using real-time reverse transcription- polymerase chain reaction (RT-PCR). A cycle threshold (Ct) value of 40 or more than was considered that the SARS-CoV-2 RNA was not present and Ct value of 37 to less than 40 required confirmation by retesting.

### Definition

Kidney disease was defined as elevated serum creatine (women: > 84 μmol/L; men: > 104 μmol/L) or positive urine protein on urine dipstick test. Proteinuria was defined as more than trace albumin on urine dipstick tests (1+, 2+, or 3+), which were collected and detected on the first morning after admission and during the observation period. Patients were included in the recovery group if the serum creatine levels return to normal and urine protein was negative at review, otherwise, patients were included in the non-recovery group.

### Statistical analysis

We performed descriptive statistics including medians and interquartile ranges (IQR) for skewed continuous measures, and proportions for categorical measures. The differences in the categorical variables were evaluated using the chi-square test or Fishers’ exact tests, and the continuous variables were compared using the Student’s t- test when normally distributed, and the Mann–Whitney test when not. Statistical analyses were performed using R software, version 3.6.1, with statistical significance set at 2-sided *P* < 0.05.

## Results

### Patients’ characteristics

Baseline characteristics, renal function measures, and biomarkers are shown in Table 1. The mean age was 64 (IQR, 51–70) years, and 59.4% of patients were men. The most frequent comorbid condition was hypertension in 48 patients (33.6%) followed by diabetes in 26 patients (18.2%). Average admission serum creatinine and discharge serum creatinine were 77 mg/dL and 68 mg/dl, respectively. The average admission eGFR and discharge eGFR were 88 mL/min/1.73 m^2^ and 93 ml/min/1.73m^2^. On admission, 83.9% (120 of 143) patients had a urine dipstick test, and 97.5% (117 of 120) patients presented with proteinuria (1+:82.5%; 2 ~ 3+: 15%). However, only 27.9% (40 of 143) patients had a urine dipstick test before discharge, and 25% (10 of 40) patients were still with proteinuria (1+: 90%; 2 ~ 3+: 10%). During hospitalization, 24 (16.8%) patients received mechanical ventilation and 3 (2.3%) patients received extracorporeal membrane oxygenation. And none of the patients had been injected with intravenous contrast dye during their hospitalization.

**Table 1:** Demographic and clinical characteristics

Clinical characteristics	No.	Summary
Age, years	143	64 (51, 70)
Male patients, No (%)	143	85 (59.4)
Fever on admission, No (%)	143	46 (32.2)
Systolic blood pressure, mmHg	143	128 (115, 141)
Diastolic blood pressure, mmHg	143	80 (71, 88)
Smoking, No (%)	143	9 (6.3)
Any comorbidity, No (%)	143	69 (48.3)
Chronic lung disease, No (%)	143	10 (7.0)
Diabetes, No (%)	143	26 (18.2)
Hypertension, No (%)	143	48 (33.6)
Tumor, No (%)	143	7 (4.9)
Blood urea nitrogen, mg/dL	143	4.9 (3.9, 6.3)
Admission SCr, mg/dL	143	77 (61, 93)
Peak SCr, mg/dL	143	84 (67, 99)
Discharge SCr, mg/dL	106	68 (60, 84)
Admission eGFR, ml/min/1.73 m2	143	88 (71, 100)
Peak eGFR, ml/min/1.73 m2	143	81 (65, 93)
Discharge eGFR, ml/min/1.73 m2	106	93 (82, 103)
Admission proteinuria	120	117 (97.5)
1+	120	99 (82.5)
2 + ~ 3+	120	18 (15.0)
Peak proteinuria	136	130 (95.5)
1+	136	107 (78.6)
2 + ~ 3+	136	23 (16.9)
discharge proteinuria	40	10 (25.0)
1+	40	9 (22.5)
2 + ~ 3+	40	1 (2.5)
Leukocyte count, × 10^9^/L	143	6.2 (4.6, 8.7)
Lymphocyte count, × 10^9^/L	143	0.8 (0.6, 1.0)
Platelet count, × 10^9^/L	143	197 (149, 269)
Hemoglobin, g/L	143	129 (121, 139)
D-dimer, mg/L	135	39 (19, 97)
hs-CRP, mg/L	134	84 (34, 134)
Admission to intensive care unit, No (%)	143	7 (0.05)
Mechanical ventilation, No (%)	143	24 (16.8)
Non-invasive, No (%)	143	22 (15.4)
Invasive, No (%)	143	6 (4.2)
ECMO, No (%)	143	3 (2.1)
Hospital length of stay, days	143	27 (21, 35)

Values for categorical variables are given as count (percentage); values for continuous variables are given as median (interquartile range). Blood pressure and laboratory data were at the time of admission. *SCr* serum creatinine; *eGFR* estimated glomerular filtration rate; *hs-CRP* high-sensitivity c-reactive protein; *ECMO* extracorporeal membrane oxygenation

### Characteristic data in the recovery vs. non-recovery group

Of the 143 patients with follow-up data, 91% (130 of 143) patients recovered from new-onset kidney disease, 9% (13 of 143) patients didn’t during a median followed-up of 4 months (Table 2). Comparison of characteristic data between the two groups is shown in Table 2. Patients in the non-recovery group were significantly older than those in the recovery group. Discharge serum creatinine was significantly higher and discharge eGFR was significantly lower in the non-recovery group than in the recovery group. Greater incidence of discharge proteinuria (66.6% versus 21.6%) was found significantly in the non-recovery group. The systolic blood pressure, comorbidities, admission serum creatinine, admission proteinuria, and leukocyte count were not significantly different between the groups.

**Table 2:** Demographic and clinical characteristics of the recovery vs. non-recovery group

	Recovery group		Non-recovery group		*p* value
Clinical characteristics	No.	Summary	No.	Summary	
Age, years	130	62 (50, 69)	13	72 (56, 82)	0.034
Male patients, No (%)	130	77 (59.2)	13	8 (61.5)	0.252
Fever on admission, No (%)	130	41 (31.5)	13	5 (38.5)	0.756
Systolic blood pressure, mmHg	130	127 (114, 141)	13	139 (121, 147)	0.180
Diastolic blood pressure, mmHg	130	80 (72, 88)	13	80 (70, 89)	0.974
Smoking, No (%)	130	7 (5.4)	13	2 (15.4)	0.191
Any comorbidity, No (%)	130	62 (47.7)	13	7 (53.8)	0.775
Chronic lung disease, No (%)	130	8 (6.2)	13	2 (15.4)	0.226
Diabetes, No (%)	130	24 (18.5)	13	2 (15.4)	> 0.999
Hypertension, No (%)	130	44 (33.8)	13	4 (30.8)	> 0.999
Tumor, No (%)	130	7 (5.4)	13	0 (0.0)	> 0.999
Blood urea nitrogen, mg/dL	130	5.0 (3.9, 6.2)	13	4.8 (3.4, 6.6)	0.763
Admission SCr, mg/dL	130	77 (62, 92)	12	83 (60, 96)	0.577
Peak SCr, mg/dL	130	84 (67, 99)	13	89 (65, 98)	0.897
Discharge SCr, mg/dL	96	66 (60, 82)	10	83 (69, 89)	0.015
Admission eGFR, ml/min/1.73m^2^	130	88 (72, 100)	12	81 (59, 91)	0.182
Peak eGFR, ml/min/1.73m^2^	130	81 (68, 93)	13	73 (48, 92)	0.478
Discharge eGFR, ml/min/1.73m^2^	96	94 (84, 103)	10	74 (66, 82)	0.003
Admission proteinuria	109	106 (97.2)	11	11 (100.0)	0.443
1+	109	91 (83.5)	11	8 (72.7)	
2 + ~ 3+	109	15 (13.7)	11	3 (27.3)	
Peak proteinuria	124	118 (95.1)	12	12 (100.0)	0.341
1+	124	99 (79.8)	12	8 (66.7)	
2 + ~ 3+	124	19 (15.3)	12	4 (33.3)	
discharge proteinuria	37	8 (21.6)	3	2 (66.6)	0.024
1+	37	8 (21.6)	3	1 (33.3)	
2 + ~ 3+	37	0 (0.0)	3	1 (33.3)	
Leukocyte count, × 10^9^/L	130	6.3 (4.6, 8.5)	13	6.2 (5.0, 9.2)	0.866
Lymphocyte count, × 10^9^/L	130	0.8 (0.6, 1.0)	13	1.0 (0.7, 1.2)	0.072
Platelet count, × 10^9^/L	130	195 (149, 263)	13	265 (142, 347)	0.161
Hemoglobin, g/L	130	129 (121, 139)	13	125 (114, 128)	0.114
D-dimer, mg/L	123	41 (19, 69)	12	31 (20, 40)	0.120
hs-CRP, mg/L	123	88 (38, 135)	11	64 (13, 107)	0.219
Admission to intensive care unit, No (%)	130	7 (0.1)	13	0 (0.0)	> 0.999
Mechanical ventilation, No (%)	130	24 (18.5)	13	0 (0.0)	0.126
Non-invasive, No (%)	130	22 (16.9)	13	0 (0.0)	0.219
Invasive, No (%)	130	6 (4.6)	13	0 (0.0)	> 0.999
ECMO, No (%)	130	3 (2.3)	13	0 (0.0)	> 0.999
Hospital length of stay, days	130	28 (21, 36)	13	21 (17, 26)	> 0.999

Values for categorical variables are given as count (percentage); values for continuous variables are given as median (interquartile range). Blood pressure and laboratory data were at the time of admission. *SCr* serum creatinine; *eGFR* estimated glomerular filtration rate; *hs-CRP* high-sensitivity c-reactive protein; *ECMO* extracorporeal membrane oxygenation

Of the 13 patients in the non-recovery group, 3 (23%) patients reviewed had slightly elevated serum creatinine and negative urine protein (Table 3). Urine dipstick in 10 (77%) patients with proteinuria during hospitalization were still reported as positive after follow-up. It was worth mentioning that in two patients, the creatinine had decreased to normal before discharge and was again elevated upon reexamination.

**Table 3:** Clinical features of 13 patients with no recovery of renal function

	Clinical characteristics					Serum creatinine				eGFR				Urine protein			
	Sex	Age	diabetes	HTN	hospital stay	Admission	Peak	Discharge	Follow-up	Admission	Peak	Discharge	Follow-up	Admission	Peak	Discharge	Follow-up
Patient 1	1	45	0	0	20	67	76	71	86		105	108	94		2	1	2
Patient 2	1	88	0	0	20	50	50		52	92	92		91	1	1		2
Patient 3	0	32	0	0	13	59	59		55	117	117		120	1	1	0	0.5
Patient 4	0	68	0	0	17	60	65	65	60	90	84	84	90	1	1		0.5
Patient 5	1	75	1	1	26	78	89	89	91	84	73	73	71	2	2		1
Patient 6	0	82	0	1	29	95	95	89	95	48	48	52	48	3	3	2	0
Patient 7	0	84	0	0	55	100	112	68	105	45	39	71	42	1	1		0
Patient 8	1	64	1	0	25	79	79		84	90	90		84	1	1		1
Patient 9	1	88	0	1	21	94	98	77	74	62	59	77	78	2	2		1
Patient 10	1	72	0	1	34	86	90	88	122	77	73	75	51	1	1		1
Patient 11	1	55	0	0	14	103	155	111	100	70	43	64	57				0
Patient 12	1	78	0	0	12	127	129	123	125	46	45	48	47	1	1		1
Patient 13	0	56	0	0	26	57	64	64	65	100	93	93	92	1	1		2
Median	1	72	0	0	21	83	89	83	86	81	73	74	78	1	1	1	

*eGFR* estimated glomerular filtration rate; *HTN* hypertension

We also summarized the medication use of both groups during hospitalization (Table 4). Most patients received antiviral therapy (95.1%) and antibiotic therapy (87.4%), and many patients received glucocorticoid therapy (58%, including at least one dose of dexamethasone in 5 mg or methylprednisolone in 20 mg, respectively). The use of antibiotics and glucocorticoids was higher in the recovery group than in the non-recovery group, although not statistically significant.

**Table 4:** Medications used during hospitalization

	All patients	Recovery group	Non-recovery group	*p* value
	(*N* = 143)	(*N* = 130)	(*N* = 13)	
RAAS inhibitors, No (%)	16 (11.2)	14 (10.8)	2 (15.4)	0.641
Antibiotics, No (%)	125 (87.4)	115 (88.5)	10 (76.9)	0.212
Antivirus, No (%)	136 (95.1)	123 (94.6)	13 (100.0)	> 0.999
Antidiabetic, No (%)	37 (25.9)	35 (26.9)	2 (15.4)	0.515
Diuretic, No (%)	18 (12.6)	18 (13.8)	0 (0.0)	0.372
Glucocorticoid, No (%)	83 (58.0)	77 (59.2)	6 (46.2)	0.390

Values for categorical variables are given as count (percentage); *RAAS* renin-angiotensin-aldosterone system

## Discussion

In the present study, we included 143 COVID-19 patients with new-onset kidney disease who were discharged alive from the hospital. During a median follow-up of 4 months, we found that 91% of COVID-19 patients recovered from new-onset kidney disease. Older age and high discharge serum creatinine were associated with non-recovery of kidney disease.

Most COVID-19 patients recovered from new-onset kidney disease 4 months after discharge. The pathophysiology and mechanisms of new-onset kidney disease in patients with COVID-19 have not been fully elucidated. Summarizing the available studies, it was found that the effects of the SARS-CoV-2 virus on the kidneys can be divided into two main aspects, the direct effects of the SARS-CoV-2 virus on the kidneys on the one hand [6, 12], and the indirect mechanisms on the kidneys due to the systemic consequences of viral infection and the effects of the virus on other distant organs on the other hand [13]. Furthermore, microscopic examination of autopsies from COVID-19 patients reported microvascular thrombosis, acute tubular necrosis, and lymphocytic infiltration of the kidneys [14–16]. In addition, although not yet fully confirmed, there is evidence that certain genetic traits in COVID-19 patients may increase susceptibility to kidney disease [17, 18]. Although kidney disease might occur in COVID-19 patients, most patients have a rapid clearance of the SARS-CoV-2 virus from the kidneys. One study examined viral nucleic acid in the urine of COVID-19 patients and found that only 4 out of 58 (6.9%) patients were positive for urine nucleic acid [19]. Thus SARS-CoV-2 virus may not cause long-term kidney damage in most patients with COVID-19. Similar results were found in the patients with COVID-19 after renal transplantation, and 9 of the 10 patients recovered successfully after a long clinical course and viral shedding [20]. A 6-month follow-up study of patients with COVID-19 found 35% of patients who experienced acute kidney injury during hospitalization had reduced eGFR (< 90 mL/min per 1.73 m^2^) at follow-up [21]. In our study, 9% of patients with new-onset kidney disease (with elevated serum creatine or positive urine protein on urine dipstick test) during the COVID-19 hospitalization didn’t recover from kidney disease. The inclusion criteria and follow-up endpoints were different between the two studies, which might be the reason for the different results. Long-term kidney recovery data should be evaluated as there was still a subset of COVID-19 patients who did not recover from kidney disease. The timely clearance of the virus, the compensatory loss of renal function after injury, and the absence of subsequent renal fibrosis might be key to the recovery mechanism. Further research is needed to better and more deeply understand the underlying mechanisms by which kidney disease occurs in COVID-19 patients and the mechanisms of recovery [22].

Older COVID-19 patients had a harder time recovering from new-onset kidney disease during hospitalization. Available results suggested that older people are more susceptible to COVID-19 infection [23] and that the older the age, the higher the mortality rate of COVID-19 infection [24, 25]. Our results showed that advanced age is a risk factor for non-recovery of kidney disease in patients with COVID-19. Indeed, the available studies suggested that the immune system appears to maintain a mild inflammatory state with advancing age and that the activation of SARS-CoV-2 invasion exaggerates the magnitude of the immune response [26]. In addition, aging is closely associated with a decline in kidney function [27]. Renal aging is characterized by a progressive increase in nephrosclerosis, loss of glomerular function, and consequently a decline in overall renal function [28]. Although to some extent, the reduction in the cortical volume of the kidney caused by nephrosclerosis is compensated by nephron hypertrophy of the remaining kidney in the medulla. However, when the patient is older than 50 years, this compensation is inadequate, and the total renal volume begins to decline [29]. In addition, elderly patients’ ability to recover from kidney damage is diminished [30]. To date, however, no study has shown that the kidneys of older people are more susceptible to, or more difficult to recover from, viral infections. Studies of potential mechanisms, identifying those factors associated with non-recovery of kidney disease and improving prognosis of kidney disease in elderly COVID-19 patients are urgently needed. Available short-term evidence suggests that older patients are more difficult to recover from kidney disease. Besides, available evidence suggests that older age is associated with worse outcomes in patients with COVID-19 [31, 32]. Therefore, renal function and proteinuria should be closely monitored and followed in elderly COVID-19 patients with new-onset kidney disease to prevent progression in clinical practice.

A high creatinine value at discharge and a positive urine dipstick test result were associated with non-recovery of kidney disease. During the hospitalization of COIVD-19 patients with new-onset kidney disease, it is necessary to closely monitor the laboratory data of renal function and urine protein dipstick test, especially before discharge. Elevated serum creatinine values and urine dipstick test positive results at discharge may be superior to help identify patients at highest risk for kidney disease and difficulty in recovery. The present study suggests that reexamination of serum creatinine and urine dipstick test before discharge may have a role in renal outcome assessment after kidney disease during hospitalization for COVID-19. Thus, to improve kidney recovery in COVID-19 patients, frequent monitoring of serum creatinine should be encouraged, especially before patients are discharged from the hospital. Regrettably, appropriate preventive surveillance strategies for kidney disease have not been widely implemented in most COVID-19 wards. Interestingly, we found that two COVID patients with new-onset kidney disease had their creatinine returned to the normal range before discharge. However, 4 months after they were discharged from the hospital, the laboratory results showed that the creatinine increased again. Additional studies are needed to address these issues. For COVID-19 patients with new-onset kidney disease during hospitalization, the reexamination of serum creatinine was recommended even if patients had normal discharged serum creatinine.

There are several limitations to this study. First, this study included a limited number of patients, all from one hospital. Second, because of the strain on medical resources caused by the sudden onset of the COVID-19 epidemic, we could not obtain comprehensive laboratory data, such as 24 h urinary protein quantification. Third, lack of data on medication exposure (e.g. non-steroid anti-inflammatory drugs) that may cause kidney injury during the follow-up period. Fourth, approximately 33% of the patients were lost to follow-up and were older than the follow-up patients. Our results may overestimate the recovery rate.

## Conclusion

Our study found that most of the new-onset kidney diseases during hospitalization of COVID-19 patients recovered 4 months after discharge. We recommend that COVID-19 patients with new-onset kidney disease be followed after discharge to evaluate kidney recovery, especially elderly patients or patients with high discharge creatinine. Larger prospective studies are needed to confirm the effectiveness of these measures.

## Supplementary Information

**Additional file 1: Table E1.** Baseline characteristics of Patients with and without follow-up data.

## Data Availability

The datasets used in this study are available from the corresponding author on reasonable request.
