# Supplementary material for: Recovery of new-onset kidney disease in COVID-19 patients discharged from hospital
Source: BMC Infect Dis. 2021 Apr 29;21:397. doi: 10.1186/s12879-021-06105-8 (PMC8083091; doi:10.1186/s12879-021-06105-8)
Supplement: Supplementary file 1 — Additional file 1: Table E1. Baseline characteristics of Patients with and without follow-up data. [file 12879_2021_6105_MOESM1_ESM.docx]

**Table E1** Baseline characteristics of Patients with and without follow-up data.

|  | All patients | | Patients with follow-up data | | Patients without follow-up data | | p |
| --- | --- | --- | --- | --- | --- | --- | --- |
| **Clinical characteristics** | No. | Summary | No. | Summary | No. | Summary |  |
| Age, years | 215 | 65 (51, 72) | 143 | 64 (51, 70) | 72 | 68 (55, 74) | 0.030 |
| Male patients, No (%) | 215 | 130 (60.5) | 143 | 85 (59.4) | 72 | 45 (62.5) | 0.737 |
| Fever on admission, No (%) | 215 | 68 (31.6) | 143 | 46 (32.2) | 72 | 22 (30.6) | 0.877 |
| Systolic blood pressure, mmHg | 215 | 130 (117.50, 142) | 143 | 128 (115, 141) | 72 | 134 (123, 144) | 0.079 |
| Diastolic blood pressure, mmHg | 215 | 80 (73, 88.50) | 143 | 80 (71, 88) | 72 | 80 (74, 90) | 0.321 |
| Smoking, No (%) | 215 | 16 (7.4) | 143 | 9 (6.3) | 72 | 7 (9.7) | 0.413 |
| Any comorbidity, No (%) | 215 | 106 (49.3) | 143 | 69 (48.3) | 72 | 37 (51.4) | 0.668 |
| Chronic lung disease, No (%) | 215 | 15 (7.0) | 143 | 10 (7.0) | 72 | 5 (6.9) | >0.999 |
| Diabetes, No (%) | 215 | 41 (19.1) | 143 | 26 (18.2) | 72 | 15 (20.8) | 0.714 |
| Hypertension, No (%) | 215 | 75 (34.9) | 143 | 48 (33.6) | 72 | 27 (37.5) | 0.649 |
| Tumor, No (%) | 215 | 11 (5.1) | 143 | 7 (4.9) | 72 | 4 (5.6) | >0.999 |
| Blood urea nitrogen, mg/dL | 215 | 4.8 (3.8, 6.3) | 143 | 4.9 (3.9, 6.3) | 72 | 4.7 (3.8, 6.5) | 0.978 |
| Admission SCr, mg/dL | 214 | 75 (61, 92) | 142 | 77 (61, 93) | 72 | 70 (60, 88) | 0.470 |
| Peak SCr, mg/dL | 215 | 83 (67, 99) | 143 | 84 (67, 99) | 72 | 81 (68, 102) | 0.832 |
| Discharge SCr, mg/dL | 162 | 68 (60, 82) | 106 | 68 (60, 84) | 56 | 69 (61, 79) | 0.914 |
| Admission eGFR, ml/min/1.73m2 | 214 | 88 (71, 99) | 142 | 88 (71, 100) | 72 | 88 (74, 98) |  |
| Peak eGFR, ml/min/1.73m2 | 215 | 82 (60, 93) | 143 | 81 (65, 93) | 72 | 84 (57, 93) |  |
| Discharge eGFR, ml/min/1.73m2 | 162 | 91 (81, 103) | 106 | 93 (82, 103) | 56 | 89 (80, 101) |  |
| Admission proteinuria | 174 | 167 (95.9) | 120 | 117 (97.5) | 54 | 50 (92.7) | 0.424 |
| 1+ | 174 | 144 (82.7) | 120 | 99 (82.5) | 54 | 45 (83.4) |  |
| 2+~3+ | 174 | 23 (13.2) | 120 | 18 (15.0) | 54 | 5 (9.3) |  |
| Peak proteinuria | 201 | 191 (95.0) | 136 | 130 (95.5) | 65 | 61 (93.8) | 0.978 |
| 1+ | 201 | 157 (78.1) | 136 | 107 (78.6) | 65 | 50 (76.9) |  |
| 2+~3+ | 201 | 34 (16.9) | 136 | 23 (16.9) | 65 | 11 (16.9) |  |
| discharge proteinuria | 57 | 15 (26.3) | 40 | 10 (25.0) | 17 | 5 (29.4) | 0.641 |
| 1+ | 57 | 13 (22.8) | 40 | 9 (22.5) | 17 | 4 (23.5) |  |
| 2+~3+ | 57 | 2 (3.5) | 40 | 1 (2.5) | 17 | 1 (5.9) |  |
| Leukocyte count, × 10⁹/L | 215 | 6.2 (4.7, 8.7) | 143 | 6.2 (4.6, 8.7) | 72 | 6.2 (5.0, 8.7) | 0.727 |
| Lymphocyte count, × 10⁹/L | 215 | 0.8 (0.6, 1.0) | 143 | 0.8 (0.6, 1.0) | 72 | 0.9 (0.6, 1.1) | 0.979 |
| Platelet count, × 10⁹/L | 215 | 197 (149, 271) | 143 | 197 (149,269) | 72 | 200 (151,281) | 0.993 |
| Hemoglobin, g/L | 215 | 129 (120, 139) | 143 | 129 (121, 139) | 72 | 128 (116, 138) | 0.215 |
| D-dimer, mg/L | 204 | 59 (27, 96) | 135 | 50 (23, 90) | 69 | 63 (30, 105) | 0.117 |
| hs-CRP, mg/L | 203 | 100 (51, 151) | 134 | 100 (50, 148) | 69 | 100 (52, 158) | 0.868 |
| Admission to intensive care unit, No (%) | 215 | 11 (0.05) | 143 | 7 (0.05) | 72 | 4 (0.06) | >0.999 |
| Mechanical ventilation, No (%) | 215 | 38 (17.7) | 143 | 24 (16.8) | 72 | 14 (19.4) | 0.705 |
| Non-invasive, No (%) | 215 | 32 (14.9) | 143 | 22 (15.4) | 72 | 10 (13.9) | 0.841 |
| Invasive, No (%) | 215 | 13 (6.0) | 143 | 6 (4.2) | 72 | 7 (9.7) | 0.132 |
| ECMO, No (%) | 215 | 4 (1.9) | 143 | 3 (2.1) | 72 | 1 (1.4) | >0.999 |
| Hospital length of stay, days | 215 | 28 (21, 39) | 143 | 27 (21, 35) | 72 | 32 (21, 40) | 0.251 |

Values for categorical variables are given as count(percentage); values for continuous variables are given as median (interquartile range). Blood pressure and laboratory data were at the time of admission. SCr, serum creatinine; eGFR, estimated glomerular filtration rate; hs-CRP, high-sensitivity c-reactive protein; ECMO, extracorporeal membrane oxygenation
